# Magnetic-free non-reciprocity based on staggered commutation

**DOI:** 10.1038/ncomms11217

**Published:** 2016-04-15

**Authors:** Negar Reiskarimian, Harish Krishnaswamy

**Affiliations:** 1 Department of Electrical Engineering, Columbia University, 1300 South West Mudd, 500 West 120th Street, New York, New York 10027, USA

## Abstract

Lorentz reciprocity is a fundamental characteristic of the vast majority of electronic and photonic structures. However, non-reciprocal components such as isolators, circulators and gyrators enable new applications ranging from radio frequencies to optical frequencies, including full-duplex wireless communication and on-chip all-optical information processing. Such components today dominantly rely on the phenomenon of Faraday rotation in magneto-optic materials. However, they are typically bulky, expensive and not suitable for insertion in a conventional integrated circuit. Here we demonstrate magnetic-free linear passive non-reciprocity based on the concept of staggered commutation. Commutation is a form of parametric modulation with very high modulation ratio. We observe that staggered commutation enables time-reversal symmetry breaking within very small dimensions (*λ*/1,250 × *λ*/1,250 in our device), resulting in a miniature radio-frequency circulator that exhibits reduced implementation complexity, very low loss, strong non-reciprocity, significantly enhanced linearity and real-time reconfigurability, and is integrated in a conventional complementary metal–oxide–semiconductor integrated circuit for the first time.

## Abstract (web summary)

Components such as isolators, circulators and gyrators are non-reciprocal in that they allow electromagnetic waves to flow in one direction but not the other. Here, the authors demonstrate a radio-frequency circulator that is compatible with and integrated in a complementary metal-oxide semiconductor integrated circuit.

## Graphical abstract

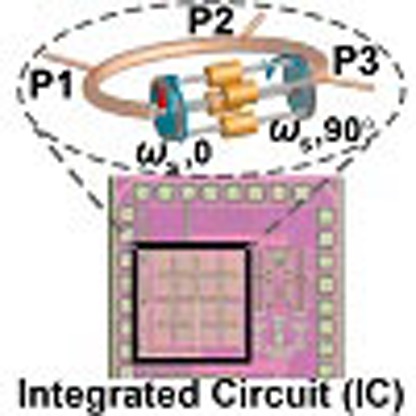

Reciprocity in electronics or, equivalently, the principle of time reversibility in optics is a fundamental property of any linear system or material described by symmetric and time-independent permittivity and permeability tensors^1^. Non-reciprocity, however, enables new applications that span radio frequencies (RF) to optical frequencies. Optical isolators are critical to on-chip all-optical information processing systems for the protection of lasers and amplifiers, and the mitigation of multipath reflections. RF circulators enable full-duplex wireless, an emerging wireless communication paradigm that has been historically considered impractical^2^, where the transmitter and the receiver operate simultaneously on the same frequency band, potentially doubling network capacity at the physical layer while offering numerous other benefits at the network layer^3^,^4^.

Non-reciprocal components today are almost exclusively realized through the magneto-optic Faraday effect (Fig. 1a–c)—the application of a magnetic field bias parallel to the direction of propagation rotates the polarization vector of light due to the different propagation velocities of left- and right-circularly polarized waves^5^. Despite significant research efforts in the optical^6^,^7^ and RF domains^8^,^9^,^10^, non-reciprocal components based on magneto-optic materials remain incompatible with complementary metal–oxide–semiconductor (CMOS) integrated circuit (IC) fabrication processes due to material incompatibilities and the need for a magnetic field bias, significantly restricting their impact.

As early as 50 years ago, magnetic-free non-reciprocity and circulators had been investigated at microwave frequencies through the use of the inherent non-reciprocal nature of active devices such as direct-current/voltage-biased transistors^11^. More recently, metamaterials with embedded active transistors have been explored at RF and microwave frequencies^12^,^13^,^14^. However, such approaches are fundamentally limited by the noise and nonlinear distortion generated by the active transistors. Indeed, for non-reciprocal components used at the front end of RF communication systems (such as circulators), and for front-end components more broadly (such as filters and diplexers), passive approaches with superior linearity and noise performance are paramount^15^,^16^. Techniques for non-reciprocity leveraging nonlinearity^17^,^18^,^19^,^20^,^21^ have also been proposed. These techniques, although potentially useful and extensively explored in optical applications, exhibit non-reciprocity over certain signal power levels only and have limited applicability to scenarios where linearity to the signal is required, such as RF communication. Lorentz reciprocity may also be broken by introducing time dependency into the material or system^1^. Techniques at optical frequencies based on electro-optic phase modulators have been investigated^22^,^23^ but require complex electro-optic modulation networks. Approaches based on spatio-temporal parametric modulation of waveguides have been investigated^24^,^25^,^26^,^27^,^28^. In such approaches, a travelling-wave modulation of the waveguide's properties produces direction-dependent mode conversion of the desired signal. In the optical domain, the size of the structure is limited by the weak electro-optic or acousto-optic effect^24^,^25^,^26^, which results in an extremely low modulation ratio (modulation ratio is defined as the ratio between the maximum and minimum values of the modulated parameter and extremely weak modulation corresponds to a modulation ratio of practically unity). At RF, varactors are able to achieve modulation ratios of around two to four, resulting in structures that are of the order of a wavelength^27^. A second disadvantage of these approaches is that the mode/frequency conversion is undesirable in some applications and necessitates the use of filters^24^ or diplexers^27^. In recent times, non-reciprocity through spatio-temporal parametric modulation of resonant rings, resulting in angular momentum biasing^29^, has been demonstrated in the acoustic domain^30^ and at RF^31^, resulting in magnetic-free non-reciprocal circulators at or well below the wavelength scale and with low loss^32^. A key challenge with these spatio-temporal parametric modulation approaches in general is that the property that enables modulation (for instance, varactors^27^,^31^ or opto-acoustic interactions^25^) often represents a nonlinearity to the signal itself, in particular when the modulation ratio is high, resulting in nonlinear distortion at higher signal levels. There has also been theoretical work on non-reciprocity and a microwave circulator based on parametric modulation of coupled resonators^33^.

Here we introduce magnetic-free, linear and passive phase non-reciprocity based on staggered commutation and a highly-miniaturized RF circulator that embeds the phase-nonreciprocal component within a ring resonator. Commutation may be seen as a form of parametric modulation with very high modulation ratio (practically infinite in our prototype), making the phase-nonreciprocal component effectively a point parametric modulator. This enables its specific placement in the ring relative to the three circulator ports such that the signal across it is suppressed for excitations from one port, yielding significantly enhanced linearity to that port. Furthermore, the need for spatio-temporal modulation in the ring is eliminated, easing modulation complexity. Implementation complexity is further eased as no frequency conversion is seen at the circulator ports. In addition, when compared with prior art employing spatio-temporal parametric modulation of waveguides^24^,^25^,^26^,^27^ or electro-optic phase modulation^22^,^23^, these concepts allow miniaturization of the unmodulated ring to deeply subwavelength scales (≈*λ*/80 in our prototype). When compared with prior art employing angular-momentum biasing in parametrically modulated resonant rings^31^,^32^, linearity enhancement is achieved due to the ability to suppress the signal across the point parametric modulator, although it is interesting to consider synergies between the approaches offering the dual benefits of both.

## Results

### Phase non-reciprocity through staggered commutation

**Figure 1:**
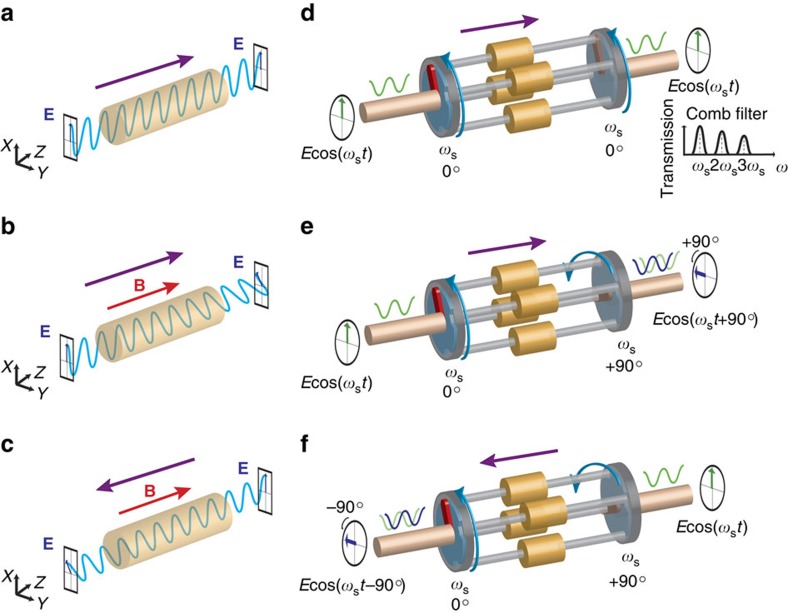
Comparison between non-reciprocity induced by the magneto-optic Faraday effect and staggered commutation. (**a**) A wave propagating in a Faraday-active magneto-optic material experiences no Faraday rotation in the absence of magnetic bias. (**b**) In the presence of magnetic bias in the positive *z* direction, a wave travelling in the positive *z* direction experiences Faraday rotation due to the difference in the propagation velocities of right-handed and left-handed circularly polarized waves, whereas (**c**) a wave travelling in the negative *z* direction experiences an opposite rotation. (**d**) A wave propagating through a commutated network with no staggering experiences no phase shift. (**e**) Staggered commutation acts as a bias that breaks time reversibility, producing a phase shift for waves travelling from left to right and (**f**) an opposite phase shift for waves travelling from right to left.

Commutated networks are a class of linear, periodically time-varying (LPTV) networks where the signal is periodically commutated through a bank of linear, time-invariant (LTI) networks (or media as in Fig. 1d). The first commutated networks relied on mechanical commutation through a rotating brush that periodically contacted a bank of capacitors^34^,^35^ to realize narrow comb filters around harmonics of the commutation frequency. More recently, electronic commutation using passive transistor-based switches has resulted in high quality-factor (*Q*) comb filters, commonly called N-path filters, which operate at RF, exhibit significantly lower noise and higher linearity when compared with active RF filters, are compatible with conventional CMOS IC technology and exhibit the potential to replace front-end off-chip surface acoustic wave filters for RF communication applications^15^,^36^,^37^,^38^,^39^,^40^. A key requirement is the availability of a high-quality switch with high ON/OFF transmission ratio (or modulation ratio when viewed as parametric modulation) and sufficient switching speed. Modern CMOS transistor switches boast ON/OFF conductance ratios as high as 1,000–100,000 (ref. 41), enough to be practically infinite from the perspective of commutated network operation, and switching speeds that enable such commutated networks to operate well into the RF frequency range and continuously improve with CMOS technology scaling. It should be emphasized that the transistors are used as reciprocal, highly linear, passive switches without direct-current bias. In other words, the switches cannot provide power gain to the desired signal by sourcing power from the modulating clock signals that control the commutation. These clocking signals that control the commutation are easily implemented in CMOS and the associated power consumption is due to the charging and discharging of parasitic capacitance in the clock path. This power consumption also reduces with CMOS technology scaling due to reducing parasitics.

An interesting property of commutated networks that we observe here is that staggering the commutation on either side of the bank of LTI networks results in time-reversal symmetry breaking and phase non-reciprocity (Fig. 1e,f). Waves travelling in the forward and reverse direction see a different ordering of the first and second commutating switches in time and experience opposite shifts in phase, essentially analogous to the magneto-optic Faraday effect. Here, the staggered commutation plays the role of the magnetic Faraday bias.

**Figure 2:**
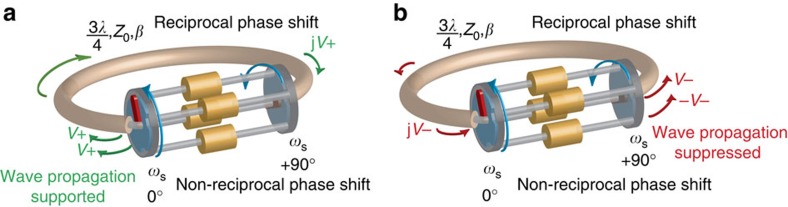
Embedding the staggered commutated network within a 3*λ*/4 transmission-line ring results in unidirectional wave propagation. (**a**) When the commutated network with +90° staggering is embedded in a 3*λ*/4 ring, then in one direction, the −270° phase delay of the ring adds to the −90° phase shift through the commutated network, enabling wave propagation. (**b**) In the other direction, the −270° phase delay adds with the +90° phase shift of the staggered commutated network, prohibiting wave propagation.

When the first and second set of switches are staggered by +90°, forward and reverse travelling waves at or near the commutation frequency experience phase shifts of +90° and −90°, respectively. When a transmission line or waveguide of length 3*λ*/4 is wrapped around the staggered commutated network, non-reciprocal wave propagation is achieved as waves may propagate in only one direction (Fig. 2a,b). In that direction, the −270° phase delay through the 3*λ*/4 ring adds with −90° phase shift of the staggered commutated network to satisfy the boundary condition, enabling wave propagation. In the other direction, the −270° phase delay adds with the +90° phase shift of the staggered commutated network to prohibit wave propagation.

**Figure 3:**
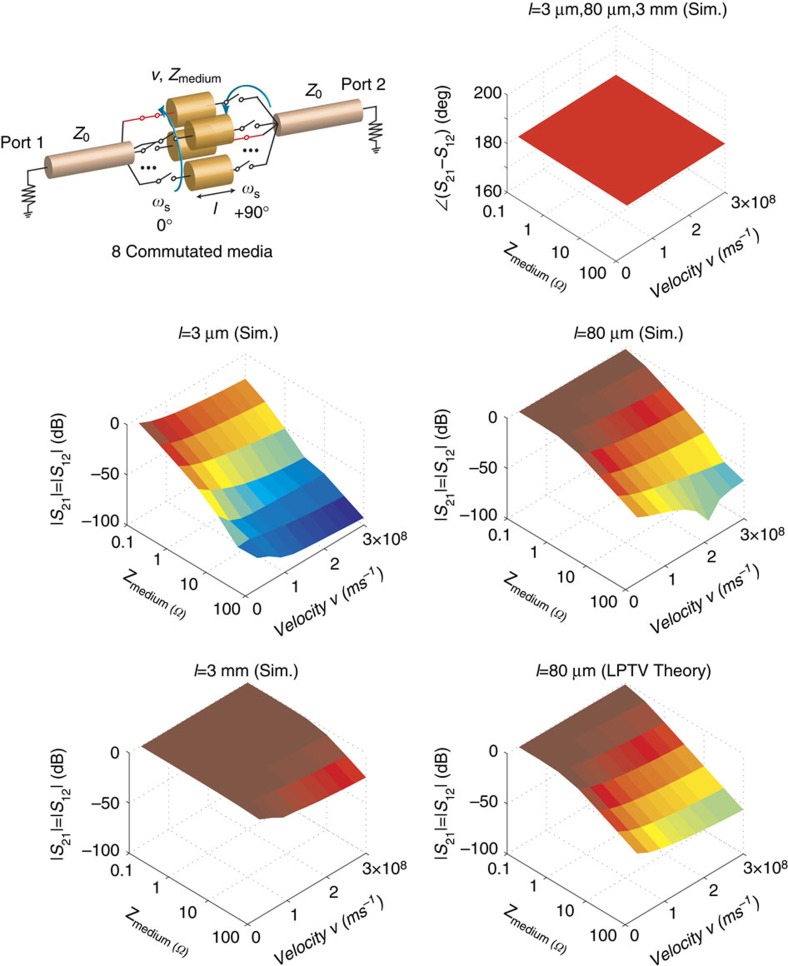
Signal transmission in a staggered commutated network. Simulated *S*-parameters of an eight-way staggered commutated network are depicted assuming electrically short transmission line media of varied length *l*, characteristic impedance *Z*_medium_ and velocity *v*. Ideal switches are commutated at 750 MHz, with each switch active for 12.5% of the time period, and the reference impedance is assumed to be 50 Ω. Theoretical calculations based on the analytical formulation presented in Supplementary Note 1, where the electrically short transmission lines are approximated by their capacitance, are also shown and agree very well with simulations. The phases of *S*_21_ and *S*_12_ are always non-reciprocal and differ by 180° for 90° staggering. The magnitudes are always reciprocal; however, for substantial transmission, high *l*, low *Z*_medium_ and low *v*, or equivalently large capacitance, are required.

Unidirectional wave propagation not only requires phase non-reciprocity but also requires (near-)perfect transmission through the commutated network. In Fig. 3, we examine the requirements on the media across which commutation is being performed. We consider electrically short transmission-line media, in line with our goal of achieving a point parametric modulator. In the depicted simulations, eight-way commutation is considered, the transmission-line characteristic impedance (*Z*_medium_), wave velocity (*v*) and length (*l*) are varied, and ideal sets of switches are used for commutation at 750 MHz frequency, with each switch active for 12.5% of the time period. With +90° staggering, phase non-reciprocity is always observed (phase of the scattering or *S*-parameters *S*_21_ and *S*_12_ at the commutation frequency always have a 180° difference). The magnitudes of *S*_21_ and *S*_12_ at the commutation frequency are always reciprocal; however, low *Z*_medium_, low velocity *v* and higher lengths *l* are required for significant signal transmission. In other words, the media must have a significant capacitance, similar to the (reciprocal) comb filter implementations described earlier, which use commutation across capacitor banks without staggering.

The need for capacitance may be intuitively understood by the fact with +90° staggering, no direct connection exists between the input and output at any instant assuming at least four paths. Therefore, a certain amount of capacitance is required for the media to store sufficient energy for subsequent transmission. A formal analysis of a commutated network with transmission-line media based on LPTV network theory is challenging. In Supplementary Note 1, with the aid of Supplementary Figs 1 and 2, we have completed an analysis of a staggered commutated network with *N* capacitors and ideal switches. As electrically short transmission lines are being considered, the approximation with capacitors is expected to be accurate. Figure 3 also depicts the magnitudes of *S*_21_ and *S*_12_ at the commutation frequency based on the exact analytical solution in Supplementary Note 1 for the case of *l*=80 μm and an excellent agreement is seen to the simulations.

The analytical formulation quantifies the capacitance condition for substantial transmission as 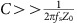 where *f*_s_ is the commutation frequency and *Z*_0_ is the reference impedance. Under this condition, at *f*_s_ for +90° staggering, the *S*-parameters of the staggered commutated network reduce to


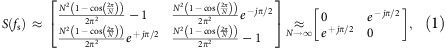


clearly showing the non-reciprocity in the phase of *S*_21_ and *S*_12_. The (reciprocal) magnitudes of *S*_21_ and *S*_12_ approach unity as *N*→∞ but even *N*=4 or *N*=8 are sufficient to achieve low loss, resulting in −1.8 and −0.45 dB transmission, respectively. The magnitudes of *S*_11_ and *S*_22_ approach 0 for *N*→∞, implying perfect impedance matching.

### A frequency up-/down-conversion and filtering-based explanation

**Figure 4:**
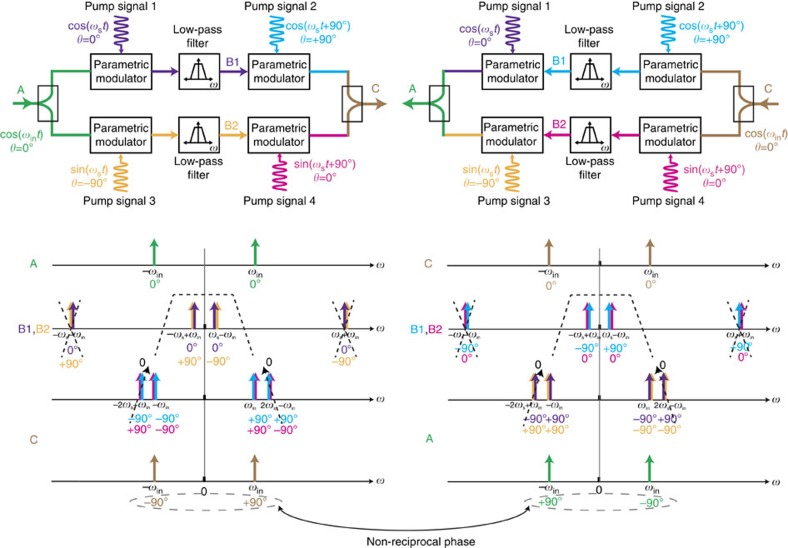
A frequency up-/down-conversion and filtering-based explanation of phase non-reciprocity in staggered commutated networks. A commutator with at least four paths can be viewed as a reciprocal in-phase and quadrature frequency up-downconverter. The capacitance of the commutated media acts as a low-pass filter that attenuates the up-converted components after the first commutation. As a result, phase non-reciprocity is seen for signals travelling in the forward and reverse directions through a staggered commutated network. It may be verified that in the absence of low-pass filtering, all frequency components at the outputs cancel, leading to zero transmission in either direction, which agrees with the theory and simulations in Fig. 3 that show very weak transmission for high *Z*_medium_, low *l* and high *v* (that is, low capacitance in the commutated media).

The behaviour may also be explained by viewing each set of commutating switches as an in-phase and quadrature reciprocal modulator that performs a frequency up-conversion and down-conversion, similar to the approach described in prior literature^33^. Indeed, such commutating transistor-based switches are regularly used as frequency converters in RF and microwave integrated electronics^42^. Figure 4 depicts a graphical view of signal propagation through the staggered commutated network in the forward and the reverse directions. In each direction, a sinusoidal input signal, cos(*ω*_in_*t*), is assumed at a frequency *ω*_in_ near the commutation frequency *ω*_s_. Each set of switches is modelled as an in-phase and quadrature reciprocal modulator that multiplies the input signal with cosine and sine versions of the pump signal at *ω*_s_. The second set of pump signals (2 and 4) are assumed to lead the first set (1 and 3) by +90°, representing the staggering. The capacitances of the media are assumed to effectively form a low-pass filter in conjunction with the source and load impedances. The frequency translation of this low-pass filtering to RF by the commutating switches is the basis for the use of unstaggered commutated networks to realize (reciprocal) comb filters^15^,^36^,^37^,^38^,^39^,^40^. This low-pass filter effect substantially attenuates the up-converted signal at *ω*_s_+*ω*_in_ and −*ω*_s_−*ω*_in_ after the first modulation. It is seen that this leads to signal transmission with non-reciprocal phase response (+90°/−90°) in the forward and reverse directions. It should be noted that although the up-converted frequency components are filtered away, this does not result in any power loss for the desired signal as the commutated media are purely capacitive. Consequently, the staggered commutated network is lossless for *N*→∞, as discussed earlier (for finite *N*, there is loss due to harmonic conversion, albeit small even for *N*=4 or *N*=8 as discussed earlier). The reader may also verify that in the absence of low-pass filtering, all frequency components at the outputs cancel and the transmission of the structure in both directions is identically zero, which agrees with the theory and simulations in Fig. 3 that show very weak tranmission for high *Z*_medium_, low *l* and high *v* (that is, low capacitance in the commutated media), and our expectation given a lack of a direct connection between the input and the output.

A comparison may be made with multi-arm optical isolators based on tandem electro-optic phase modulation^22^, where back-to-back phase modulation with quarter-wavelength optical delays in between results in non-reciprocity. In our approach, commutation is essentially amplitude modulation with very high modulation ratio. Staggered commutation results in phase non-reciprocity and the filtering effect that is unique to commutation across media with appreciable capacitance eliminates the need for quarter-wavelength delays, allowing the phase non-reciprocity to be achieved within dimensions as small as *λ*/1,250 × *λ*/1,250 in our device.

### A highly-miniaturized RF circulator with enhanced linearity

**Figure 5:**
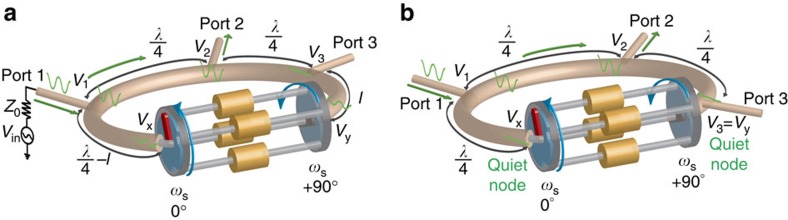
Circulator architecture. (**a**) A 3-port circulator may be realized by adding ports at three points along the ring as long as the circumferential spacing between the ports is *λ*/4. The *S*-parameters of such a structure match that of an ideal three-port circulator and are independent of *l*, the circumferential distance between port 3 and the staggered commutated network. (**b**) However, setting *l* to 0 suppresses the voltages across the commutated network for excitations at port 1, significantly enhancing the linearity of the structure to port 1 excitations.

A three-port circulator matched at all ports to a reference impedance of *Z*_0_ may be realized by contacting the 3*λ*/4 transmission line (also of characteristic impedance *Z*_0_) at three points as long as the three ports are spaced *λ*/4 apart along the ring circumference, as shown in Fig. 5a. To analyse this structure, we consider the case of a voltage excitation *V*_in_ at port 1. Using conventional microwave circuit analysis techniques along with the *S*-parameters of the staggered commutated network in equation (1) for the case of *N*→∞, the various node voltages can be determined to be


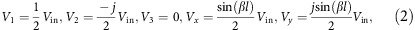


where *V*_1_, *V*_2_ and *V*_3_ are the three port voltages, *V*_*x*_ and *V*_*y*_ are the voltages on the left and right sides of the staggered commutated network, *β* is the propagation constant of the 3*λ*/4 ring and *l* is the circumferential distance between port 3 and the commutated network. Based on these node voltages and by repeating the calculations with excitations at the other two ports, the *S*-parameter matrix of the structure can be derived to be:


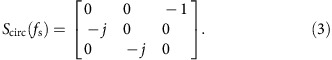


This matches the *S*-parameters of an ideal 3-port circulator. It is interesting to note that the *S*-parameter matrix does not depend on *l*, meaning that the position of the staggered commutated network relative to ports 1 and 3 does not have an impact on *S*_circ_. However, the voltages seen at the two ends of the staggered commutated network (*V*_*x*_ and *V*_*y*_) when port 1 is excited are functions of *l* in equation (2). Interestingly, these voltages become 0 when *l* is 0. This is a direct consequence of the fact that when *l* is 0, *V*_*y*_ coincides with port 3 and naturally remains quiet for excitations at port 1 due to the isolation of the circulator (Fig. 5b). The *S*-parameters of the staggered commutated network force *V*_*x*_ and *V*_*y*_ to have the same magnitude, making *V*_*x*_ also a quiet node (Fig. 5b). As a result, voltage swings across the point parametric modulator are suppressed, yielding very high linearity to excitations at port 1.

A more comprehensive analysis of the circulator may be performed using the *S*-parameters of the staggered commutated network in equation (1) for the case of finite *N*. Interestingly, for *l*=0, we once again arrive at the ideal *S*-parameter matrix of equation (3) and *V*_*x*_=*V*_*y*_=0 for excitations at port 1. This implies that when port 3 coincides with *V*_*y*_, the presence of a finite number of commutated paths will not limit circulator performance. In the presence of finite switch ON resistance, however, there will be finite voltage swing across the commutating switches for excitations at port 1, leading to finite linearity. However, this linearity to port 1 excitations will be vastly superior to the linearity to excitations at the other ports that do not enjoy this suppression mechanism.

**Figure 6:**
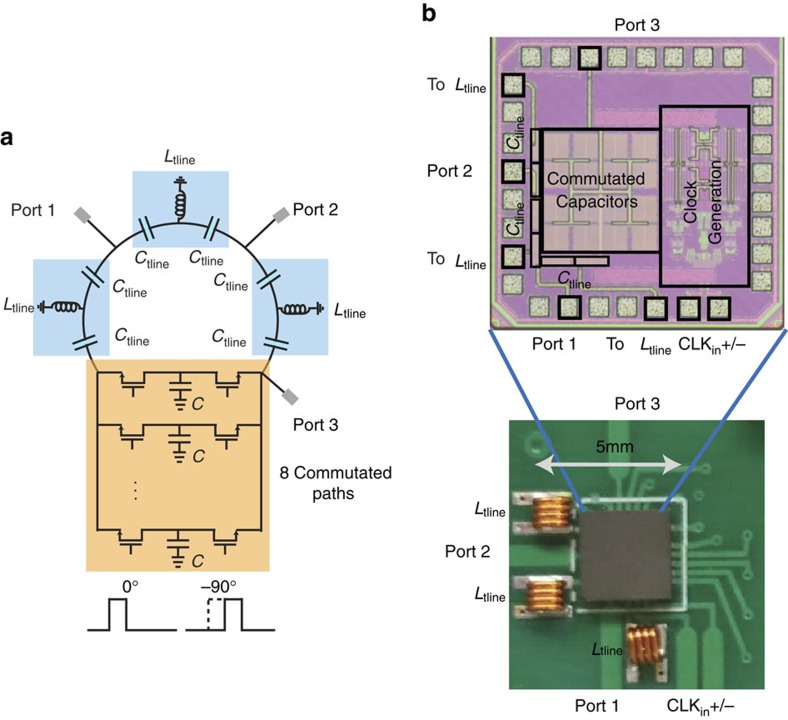
RF CMOS IC implementation of the circulator. (**a**) A simplified circuit diagram of the circulator is shown. Electronic commutation across a bank of *N*=8 capacitors is performed using reciprocal, passive transistor-based switches without direct-current bias. The staggered commutated network enables miniaturization of the unmodulated 3*λ*/4 ring using three C-L-C sections. (**b**) The microphotograph of the fabricated IC is shown along with a close-up photograph of the fabricated printed circuit board with the IC housed in a quad-flat no-leads (QFN) package and interfaced with the off-chip inductors. The largest dimension of the prototype is 5 mm or *λ*/80 at the operating frequency of 750 MHz.

We constructed a prototype at RF using conventional 65 nm CMOS IC technology to implement the electronic commutation across a bank of *N*=8 capacitors, and three lumped C-L-C networks to miniaturize the 3*λ*/4 transmission line ring (Fig. 6). Miniaturization is eased by the fact that the 3*λ*/4 ring is unmodulated, resulting in an overall structure that has a maximum dimension of ≈*λ*/80 at the operating frequency of 750 MHz. The capacitors of the C-L-C networks are also incorporated in the IC, leaving the three inductors as the only off-chip components. The commutated capacitors are chosen to be 26 pF each, roughly six times 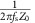 (*Z*_0_=50 Ω), and are each realized on chip as a pair of 80 μm × 80 μm metal–insulator–metal capacitors. The total size of the staggered commutated network is ∼320 μm × 320 μm (*λ*/1,250 × *λ*/1,250). The control signals for the staggered commutation are generated using on-chip clock generation circuitry that is described in additional detail in the Methods section.

### Experimental results

**Figure 7:**
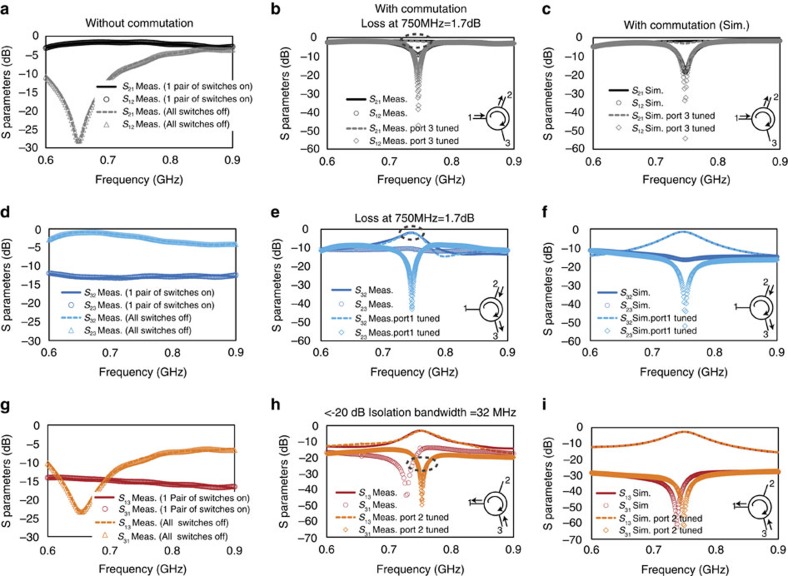
Circulator *S*-parameter measurements. Measured circulator *S*-parameters (**a**), (**d**) and (**g**) without commutation and (**b**), (**e**) and (**h**) with commutation are shown, as well as (**c**), (**f**) and (**i**) simulated *S*-parameters that show an excellent match to the measurements. Under staggered commutation for clockwise circulation, low loss transmission in the circulation direction (*S*_21_, *S*_32_ and *S*_13_ are −1.7, −1.7 and −3.3 dB, respectively) and an order-of-magnitude isolation in the reverse direction (*S*_12_, *S*_23_ and *S*_31_ are −9.6, −10.4 and −17.4 dB, respectively) are measured at the commutation frequency of 750 MHz. When the third port is slightly tuned, non-reciprocity of 40–50 dB is measured in *S*_12_, *S*_23_ and *S*_31_ at 750 MHz with negligible impact on transmission in the circulation direction. The −20 dB isolation bandwidth in *S*_31_ after tuning is 32 MHz or 4.3%.

*S*-parameter measurements of the three-port circulator were performed using a measurement setup described in the Methods section. In the absence of commutation, with a pair of transistor switches on either side of the capacitor bank permanently closed, the circuit is perfectly reciprocal (Fig. 7a,d,g). In this configuration the high reciprocal isolations from port 3 to ports 1 and 2 are seen because port 3 is shunted to ground by one of the 26-pF capacitors. The quarter-wave transmission lines from port 1 to the staggered commutated network and from port 2 to port 3 transform this near-short-circuit impedance to open circuits at ports 1 and 2, effectively disconnecting port 3 from the rest of the circuit and resulting in a reciprocal structure that exhibits low-loss transmission between port 1 and port 2. Similarly, another reciprocal configuration without commutation is depicted, where all switches are permanently open. Here, simple circuit analysis reveals that port 1 is effectively disconnected from the rest of the circuit, which now exhibits low-loss reciprocal transmission between ports 2 and 3.

Under commutation at a frequency of 750 MHz with staggering for clockwise circulation, strong non-reciprocity is measured (Fig. 7b,e,h) with low-loss transmission in the direction of circulation (*S*_21_, *S*_32_ and *S*_13_ are −1.7, −1.7 and −3.3 dB, respectively, at 750 MHz) and strong isolation in the reverse direction (*S*_12_, *S*_23_ and *S*_31_ are −9.6, −10.4 and −17.4 dB, respectively, at 750 MHz). This represents, on an average, an order of magnitude of non-reciprocity between any two ports and is limited by the imperfect impedance matching at the third port (as is the case with all circulators) caused due to parasitics at the chip-package-board interfaces. If the impedance at the third port is slightly tuned, non-reciprocity of 40–50 dB or four to five orders of magnitude is seen in *S*_12_, *S*_23_ and *S*_31_ at 750 MHz with negligible impact on transmission in the circulation direction. It should be noted that independent tuning is required at the three ports to achieve very high non-reciprocity in all paths, given the inherent asymmetry in the circulator structure. Figure 7c,f,i depict the simulated *S*-parameters under commutation, demonstrating an excellent match to the measurements.

Although the analysis in this study has restricted itself to response at the commutation frequency, it is noteworthy that a filtering profile is observed in *S*_32_ and *S*_13_ across frequency due to the comb-filter functionality inherent to commutation across a capacitor bank. The LPTV analysis in Supplementary Note 1 may be extended to determine the response of the staggered commutated network and the circulator across frequency, and confirms this observed (and simulated) filtering profile.

**Figure 8:**
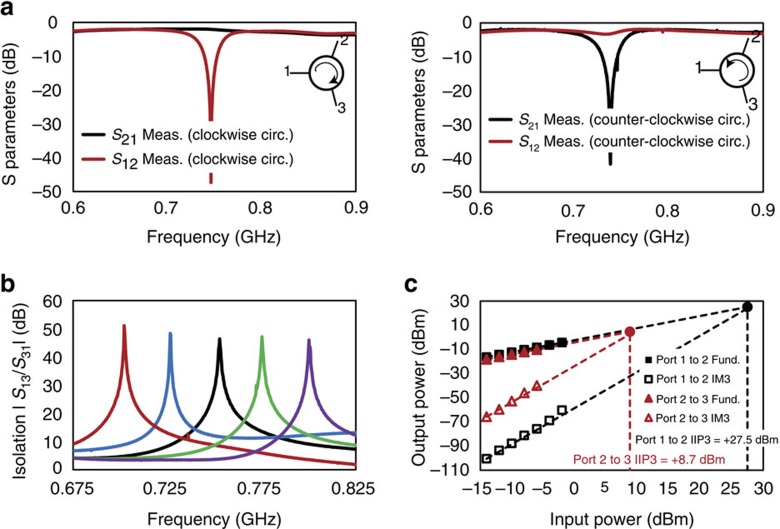
Experimental evidence of reconfigurability and enhanced linearity to port 1 excitations. (**a**) The direction of circulation can be altered by changing the staggering between +90° and −90°. (**b**) The frequency of operation of the circulator can be tuned by changing the commutation frequency within the limits dictated by the bandwidth of the 3*λ*/4 transmission-line ring. Here we present the non-reciprocity in *S*_31_ across different commutation frequencies ranging from 700 to 800 MHz. In each case, tuning of the port 2 impedance is exploited to achieve 40–50 dB isolation at the commutation frequency. (**c**) Measured two-tone linearity for transmission from port 1 to port 2 and from port 2 to port 3 are shown when configured for clockwise circulation. Nonlinear systems exhibit intermodulation distortion products when excited with two sinusoidal signals. The input-referred third-order intercept point (IIP3) represents the (extrapolated) input power of each of the two tones at which the third-order intermodulation products (IM3) at the output are as powerful as the fundamental signals. The IIP3 for transmission from port 1 to port 2 is +27.5 dBm (≈560 mW), nearly two orders of magnitude higher than that from port 2 to port 3 (+8.7 dBm or 7.4 mW), owing to the suppression of the signal across the point parametric modulator for port 1 excitations.

Another unique feature of the fabricated prototype is its real-time reconfigurability. By changing the staggering between +90° and −90°, the direction of circulation can be altered, as is seen in Fig. 8a. The frequency of operation of the circulator can also be tuned by changing the frequency of commutation within the limits dictated by the bandwidth of the 3*λ*/4 transmission line ring. In Fig. 8b, four to five orders of magnitude (40–50 dB) of non-reciprocity in *S*_31_ is maintained across 700–800 MHz.

In Fig. 8c, we present experimental evidence of the enhanced linearity to excitations at port 1. Two-tone intermodulation distortion tests were performed on the prototype for transmission from port 1 to port 2, and from port 2 to port 3. The input-referred third-order intercept point (IIP3) for transmission from port 1 to port 2 is +27.5 dBm (≈560 mW), a remarkable number for a 65nm CMOS IC implementation and nearly two orders of magnitude higher than that from port 2 to port 3 (+8.7 dBm or 7.4 mW) due to the suppression of the signal across the point parametric modulator. Aside from intermodulation distortion caused due to third-order nonlinearity, other spurious effects to consider include image signals due to quadrature mismatch and modulation feedthrough. The level of the measured spurious image signals produced due to quadrature mismatch for port 1 to port 2 transmission is 51 dB below the main signal. This level of image rejection is high enough so that it is not a serious issue when compared with the third-order intermodulation distortion produced for reasonable port 1 power levels. The measured modulation feedthrough at port 2 is at −57 dBm. Although these levels are already quite low, they can be cancelled further using integrated calibration circuits that are commonly implemented in CMOS RF ICs.

Passive LTI systems have a noise figure (NF) that is equal to their loss. Passive LPTV systems can exhibit noise folding. The measured NF for port 2 to port 3 transmission is 4 dB, higher than the 1.7dB loss. Simulations indicate that 2 dB degradation arises from modulation path phase noise due to a poor implementation of the modulation path phase shifter. A better implementation of the modulation path phase shifter restores the NF to 2 dB in simulation, close to the 1.7 dB loss level and indicative of only minor NF degradation due to harmonic noise folding.

## Discussion

The ability to integrate magnetic-free passive linear non-reciprocal components in CMOS has the potential to revolutionize RF communications. The CMOS circulator described in this study exhibits extremely low insertion loss (<2 dB) and strong non-reciprocity, is compact and features reconfiguration capabilities. Furthermore, the enhanced linearity to port 1 is particularly useful for full-duplex or simultaneous transmit-and-receive communication and radar applications^3^,^4^, where a high-power transmitter (port 1) and a highly sensitive receiver (port 3) operate simultaneously at the same frequency, and must be interfaced with a shared antenna (port 2). In such a configuration, *S*_21_ (transmitter to antenna loss), *S*_32_ (antenna to receiver loss) and *S*_31_ (transmitter to receiver isolation) are the most important performance metrics, where our device performs particularly well. Full-duplex communication is drawing significant interest for emerging 5G communication networks^43^ due to its potential to double network capacity compared with half-duplex communication at the physical layer while offering numerous other benefits at the network layer. There has been active research on fully integrated CMOS transceiver ICs supporting full duplex^44^,^45^,^46^,^47^,^48^. The ability to include the circulator with the transceiver on the same CMOS IC would significantly reduce the cost and form factor, and enhance the performance of full-duplex systems. The filtering profile in *S*_32_ is also very useful in protecting the highly sensitive receiver from interference signals outside the frequency band of operation.

For such applications, the ability to reconfigure the device between reciprocal and non-reciprocal operation is also very useful. We demonstrated reconfigurable modes of operation in the absence of commutation where the structure becomes reciprocal with low-loss transmission between the antenna (port 2) and the transmitter (port 1), and the antenna and the receiver (port 3). In other words, the prototype can be reconfigured between operation as a non-reciprocal circulator for full-duplex communication and a reciprocal low-loss transmit/receive switch for half-duplex communication.

Although the design of this prototype is deliberately asymmetric with respect to the three ports to prioritize linearity to port 1 excitations for communication applications, a symmetric design may also be envisioned where a non-reciprocal phase component is incorporated in all three arms, that is, between all three ports. Such a symmetric circulator structure would enable extension of the concept to non-reciprocal metamaterials that support topologically protected wave propagation modes^49^.

The bandwidth of the circulator's isolation is limited by the matching between the frequency responses of the 3*λ*/4 ring and the staggered commutated network. Currently, the −20 dB isolation bandwidth in *S*_31_ after tuning is 32 MHz or 4.3% of the operating frequency, useful for commercial cellular LTE and WiFi applications. Dispersion engineering techniques to match their responses over a wider frequency range, thus enhancing the isolation amount versus bandwidth trade-off, represent an interesting topic for future research.

Commutated networks are known to have response at harmonics of the commutation frequency. Response at harmonics can lead to susceptibility to interference near harmonic frequencies and also leads to noise folding. Although we have seen that the effect of noise folding on the NF is very small in this circulator structure, a formal analysis of the harmonic response and noise of the overall circulator structure, as well as techniques to suppress the same, also represent an interesting topic for future investigation.

It is also interesting to consider the application of these concepts to other domains where a high-quality switch is available, such as optical waves. Compact optical switches with one to two orders of magnitude ON/OFF transmission ratio^50^,^51^ open the door to optical non-reciprocity and isolation through commutation-based parametric modulation. The nanosecond-scale switching speed implies GHz-range commutation frequencies, much smaller than the optical carrier frequency, which can be accomodated by commutating across high-Q optical filters that eliminate one of the modulation sidebands, similar to the low-pass filter effect used in our prototype.

## Methods

### IC implementation details

A block diagram of the IC and component values are provided in Supplementary Fig. 3 and Supplementary Table 1, respectively. The circulator consists of three lumped C-L-C sections that miniaturize the 3*λ*/4 transmission line and a staggered electronically commutated network of eight capacitors. All components, except for the three inductors, are integrated on the CMOS IC. The switches of the commutated network are implemented using 65 nm CMOS transistors and are driven with two sets of eight non-overlapping clock signals with 12.5% duty cycle. These clock signals are generated from two differential (0/180°) input clocks that run at four times the desired commutation frequency. A divide-by-2 frequency-divider circuit generates four quadrature clocks with 0°/90°/180°/270° phase relationship. These four clock signals drive two parallel paths for the two sets of switches. One of the paths features a programmable phase shifter that allows for arbitrary staggering between the two commutating switch sets. This enables switching between +90° and −90° staggering, which allows dynamic reconfiguration of the circulation direction. The phase shifter also allows for fine tuning of the staggered phase shift, to optimize the transmission loss in the circulation direction and isolation in the reverse direction. This feature has been exploited in the measurements shown in the main text. After phase shifting, another divide-by-2 circuit and a non-overlapping 12.5% duty-cycle clock generation circuit create the clock signals that control the commutating transistor switches.

### Experimental setups

Diagrams of the experimental setups are provided in Supplementary Figs 4 and 5, respectively. A list of the equipment used in the two setups is provided in Supplementary Table 2. A 180° hybrid is used to generate two differential (0°/180°) signals from a signal generator to drive the clock inputs of the implemented circulator. A voltage bias signal is added to the clock signals through bias tees to drive the clock inputs. A two-port vector network analyser is used to measure the *S*-parameters of the circulator two ports at a time, whereas the third port is terminated with a variable impedance tuner to tune the port impedance. For the input-referred third-order intercept point linearity test, a two-way power combiner is used to combine two sinusoidal signals generated from two additional signal generators and feed the input port under consideration. A spectrum analyser is used to monitor the fundamental signals and the third-order intermodulation distortion products of the circulator at the output port under consideration, whereas the third port is terminated with a 50-Ω termination.

## Additional information

**How to cite this article:** Reiskarimian, N. & Krishnaswamy, H. Magnetic-free non-reciprocity based on staggered commutation. *Nat. Commun.* 7:11217 doi: 10.1038/ncomms11217 (2016).

## Supplementary Material

Supplementary Information Supplementary Figures 1-5, Supplementary Tables 1-2, Supplementary Note 1 and Supplementary References.
