# Supplementary material for: Magnetic-free non-reciprocity based on staggered commutation
Source: Nat Commun. 2016 Apr 15;7:11217. doi: 10.1038/ncomms11217 (PMC4835534; doi:10.1038/ncomms11217)
Supplement: Supplementary Information — Supplementary Figures 1-5, Supplementary Tables 1-2, Supplementary Note 1 and Supplementary References. [file ncomms11217-s1.pdf]

Supplementary Figures

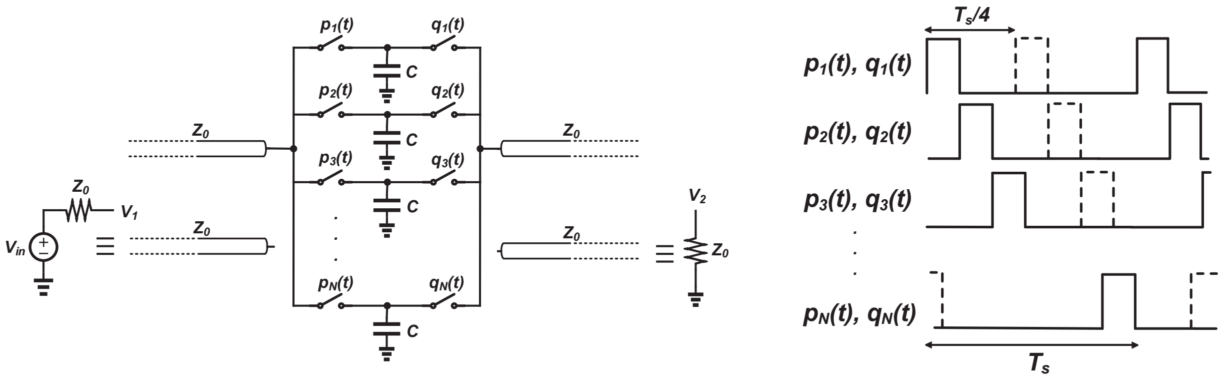

Supplementary Figure 1: A staggered commutated network of N capacitors, each of value C.

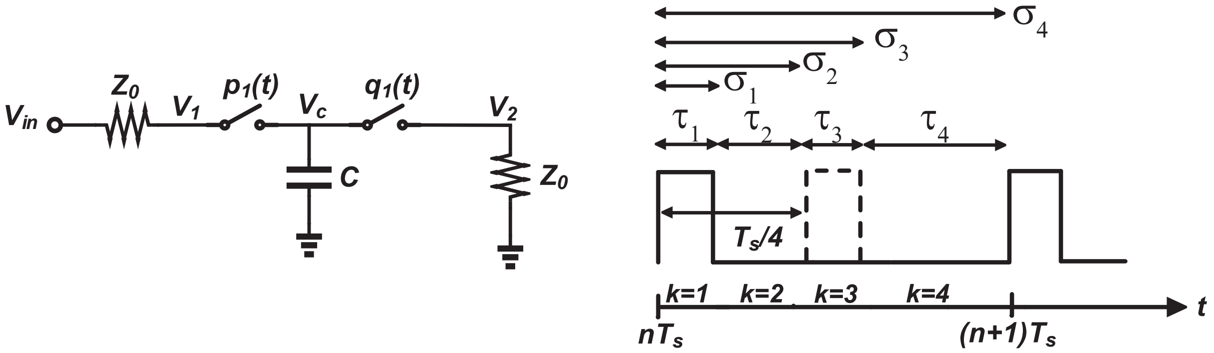

Supplementary Figure 2: Single-state kernel and timing diagram for the control signals.

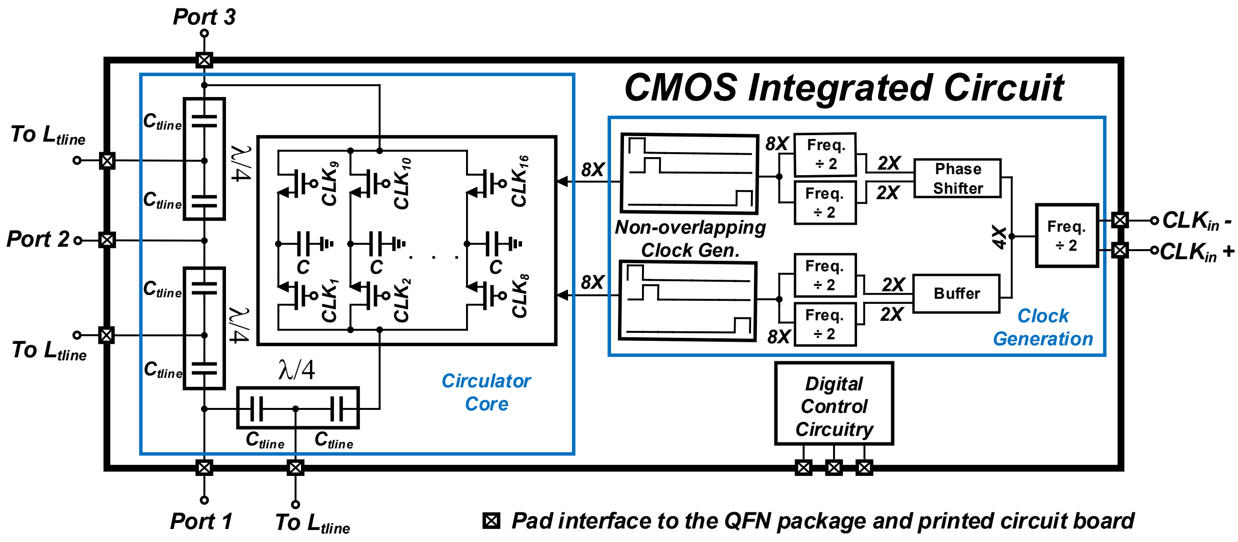

Supplementary Figure 3: CMOS integrated-circuit (IC) implementation of the circulator.

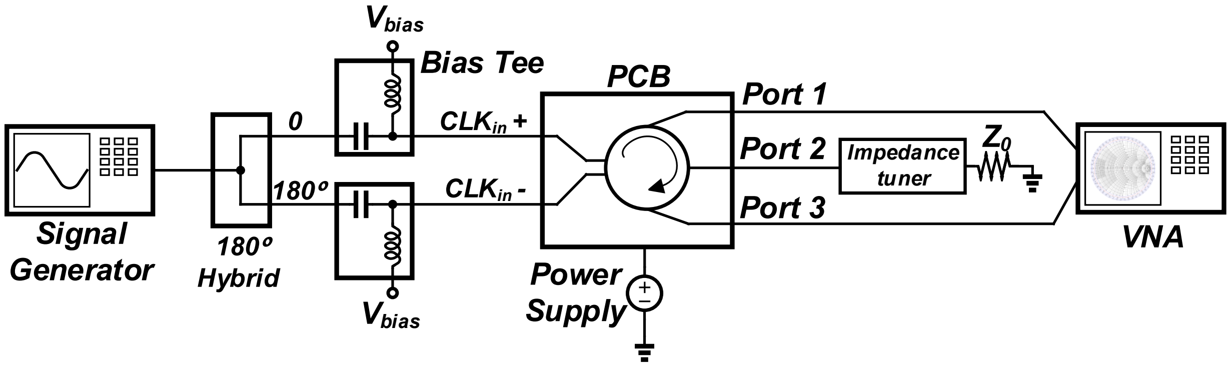

Supplementary Figure 4: Experimental setup for S-parameter measurements.

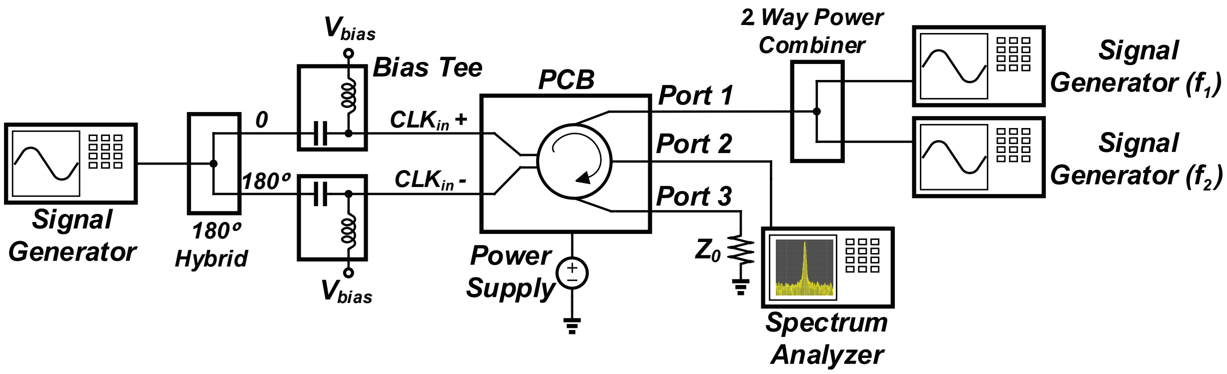

Supplementary Figure 5: Experimental setup for the input-referred third-order intercept point (IIP3) linearity test.

Supplementary Tables

Supplementary Table 1: Values of the components used in the integrated circulator: the inductor is an off-the-shelf surface-mount component, while the capacitors are implemented on the integrated circuit.

**Component**	Value
L_{tline} **(Coilcraft 0806SQ-8N9)**	8.9nH
C	26pF
C_{tline}	4.1pF

Supplementary Table 2: List of equipment used in the experimental setups.

**Equipment**	Model
**Vector Network Analyzer**	Anritsu 37397E
**Spectrum Analyzer**	Agilent E4448A
**Signal Generators**	Agilent E8257D<br>HP 83712B<br>Anritsu MG3697C
**Power Supplies**	Agilent E3631A<br>Agilent E3632A<br>Agilent U8002A
**Impedance Tuner**	Maury Microwave MST982VN
**Bias Tees**	Mini-Circuits ZX85-12G+
180° **Hybrid**	RF Lambda RFHB02G08GPI
**2-Way Power Combiner**	RF Lambda RFHB75M02GPI

Supplementary Note 1: Linear Periodically-Time-Varying Network Theoretic Analysis of Staggered Commutated Networks

In this supplementary note, we provide an analysis of the staggered commutated network based on linear periodically-time-varying (LPTV) network theory [1,2].

As mentioned in the main text, LPTV analysis of commutation across an array of transmission lines is analytically challenging. Here, we consider an array of capacitors with commutating switches on both sides (Supplementary Figure 1). Since we are interested in electrically-small transmission lines enabling effectively a point parametric modulator, the approximation of the transmission lines with a capacitance is expected to be accurate. An array of N capacitors is considered, each of value C, and the switches are controlled by the signals denoted by $p_i(t)$ and $q_i(t)$ in Supplementary Figure 1. A high level of the control signals denotes that the corresponding switch is in the ON state, while a low level denotes the OFF state. While the analysis considered would generally apply to any commutated network (electronic, optical or otherwise), these control signals are highly representative of the clock signals that are generated on our complementary metal-oxide-semiconductor (CMOS) IC to control the transistor-based switches. Each switch is ON for a $\frac{1}{N}$ fraction of the commutation period T_s. The switches are assumed to be ideal with infinite modulation ratio - in other words, in the ON state, the switches have zero resistance or perfect transmission, and in the OFF state, the switches have infinite resistance or perfect (reciprocal) isolation. Capacitive parasitics in the switches are ignored under the assumption that switching speed is significantly higher than the commutation frequency. The $q_i(t)$ control signals are delayed

by $T_s/4$ compared to the $p_i(t)$ signals representing staggered commutation by -90°. An input voltage source V_{in} and source resistance of value Z_0 models the feed transmission line, and a load resistance of Z_0 models the load transmission line.

In linear periodically-time-varying (LPTV) systems, the input and output spectra are related as [1,2]:

$$Y(f) = \sum_{n=-\infty}^{+\infty} H_n(f) X(f - nf_s), \tag{1}$$

where $X(f)$ and $Y(f)$ are the input and output spectra, respectively, and $f_s = \frac{1}{T_s}$ is the periodicity of time variance. The output is a summation of an infinite number of frequency-shifted versions of the input signal, each undergoing a different harmonic transfer function (HTF) denoted by an index n for nf_s amount of frequency shift.

The circuit in Supplementary Figure 1 is an LPTV system where the capacitor voltages represent the states of the system, and the voltages V_1 and V_2 are combinations of the circuit states. Furthermore, the control signal sets $p_i(t)$ and $q_i(t)$ are *polyphase* signals, meaning the control signal within each set have the same duty cycle (ratio of ON period to total period), start at regularly space intervals within the period time and are non-overlapping. The non-overlapping nature implies that there is no instant of time when two capacitors are connected to each other, rendering the circuit states independent of each other. Such a circuit can be analyzed by decomposing the circuit into single-state kernels, and performing LPTV analysis on the kernel to determine its HTFs. The

HTFs are then combined across the kernels to find the overall HTFs to V_2 or V_1. The reader is directed to prior literature[2] for additional details on this analytical approach.

The single-state kernel is shown in Supplementary Figure 2. Furthermore, based on the timing of the control signals, a single period can be divided into the sub-periods τ_1, τ_2, τ_3 and τ_4. Within each sub-period, the kernel has a valid LTI state-space description. The n-th period is shown in Supplementary Figure 2. The durations of the sub-periods are given by:

$$\tau_1 = \tau_3 = \frac{T_s}{N}, \tau_2 = \frac{T_s}{4} - \frac{T_s}{N}, \tau_4 = T_s - \tau_1 - \tau_2 - \tau_3, \sigma_0 = 0, \sigma_k = \sum_{i=1}^{k} \tau_i. \tag{2}$$

It should be noted that we are implicitly assuming $N \geq 4$ in the timing diagram of Supplementary Figure 2 so that $p_i(t)$ and $q_i(t)$ don't overlap with each other. As mentioned earlier, in each sub-period marked in Supplementary Figure 2, the kernel can be analyzed using LTI theory. For example, in the first sub-period, we have:

$$\frac{dv_c}{dt} = A_1 v_c(t) + B_1 v_{in}(t),\ nT_s \leq t < nT_s + \sigma_1, \tag{3}$$

$$A_1 = \frac{-1}{Z_0 C},\ B_1 = \frac{1}{Z_0 C}. \tag{4}$$

It should be noted that this equation only holds for $nT_s < t < nT_s + \frac{T_s}{N}$. A similar LTI

differential equation can be written for other sub-periods as well with $A_2 = 0, A_3 = \frac{-1}{Z_0 C}, A_4 = 0$ and $B_2 = 0, B_3 = 0, B_4 = 0$. We now define $v_{c,k}(t)$ as a signal that is equal to $v_c(t)$ within the k-th sub-period and zero otherwise. In other words, $v_{c,k}(t) = v_c(t) \times w_k(t)$ where $w_k(t)$ is a windowing function given by $w_k(t) = 1, nT_s + \sigma_{k-1} \leq t < nT_s + \sigma_k$ and 0 elsewhere. Accounting for the boundary conditions at the beginning and end of each sub-period, we get:

$$\frac{dv_{c,k}}{dt} = A_k v_{c,k}(t) + B_k v_{in,k}(t) + \sum_{n=-\infty}^{\infty} (v_c(t)\delta(t - nT_s - \sigma_{k-1}) - v_c(t)\delta(t - nT_s - \sigma_k)), -\infty < t < \infty. \tag{5}$$

It should be noted that $v_{in,k}(t) = v_{in}(t) \times w_k(t)$. Since (5) is valid over all time, a Fourier transform may be evaluated as below.

$$V_{c,k}(f) = \sum_{n=-\infty}^{\infty} H_{n,k}^{Vc,kernel}(f) V_{in}(f - nf_s), \tag{6}$$

where

$$H_{n,k}^{Vc,kernel}(f) = \frac{1}{j2\pi f - A_k}(B_k \frac{1 - e^{-j2\pi n f_s \tau_k}}{j2\pi n} e^{-j2\pi n f_s \sigma_{k-1}}$$
$$+ f_s G_{k-1}(f - nf_s) e^{-j2\pi n f_s \sigma_{k-1}} - f_s G_k(f - nf_s) e^{-j2\pi n f_s \sigma_k}). \tag{7}$$

The superscript adopted in the notation clarifies that we are attempting to determine the

harmonic transfer function to V_c in the single-state kernel. In this equation, the G_k functions are the switching-moment transfer functions of the system at the switching moments $t = nT_s + \sigma_k$. Additional details related to the derivation of (7) may be found in prior literature[2]. Note that we are endeavoring to derive the scattering matrix of the staggered commutated network, and are only interested in the H_0 responses, and not in any frequency translations of the input. Furthermore, the output voltage V_2 in the single-state kernel (shown in Supplementary Figure 2) is equal to V_c during the third interval and is 0 otherwise, resulting in $H_0^{V2,kernel} = H_{0,3}^{Vc,kernel}$. The $G_k(f)$s in (7) can be evaluated by following the steps delineated in prior literature [2] to obtain the generic form:

$$G_k(f) = \frac{\beta_k}{e^{j2\pi f T_s} - e^{-2\pi f_C(\tau_1+\tau_3)}}, \tag{8}$$

where $f_C = \frac{1}{2\pi Z_0 C}$ and

$$\begin{aligned}\beta_2 &= \frac{e^{j2\pi f(\tau_3+\tau_4)}}{1+\frac{jf}{f_C}}[e^{j2\pi f\tau_1} - e^{-2\pi f_C \tau_1}],\\ \beta_3 &= \frac{e^{j2\pi f\tau_4} e^{-2\pi f_C \tau_3}}{1+\frac{jf}{f_C}}[e^{j2\pi f\tau_1} - e^{-2\pi f_C \tau_1}].\end{aligned} \tag{9}$$

Note that only β_2 and β_3 are of interest since $H_n^{V2,kernel} = H_{n,3}^{Vc,kernel}$. Substituting for A_k, B_k and G_k in (7), we derive:

$$H_0^{V2,kernel}(f) = \frac{f_s e^{j2\pi f(\frac{T_s(N-1)}{N} - \frac{T_s}{4})}}{2\pi f_C(1+\frac{jf}{f_C})^2} \times \frac{(e^{j2\pi f\frac{T_s}{N}} - e^{-2\pi f_C\frac{T_s}{N}})^2}{e^{j2\pi f T_s} - e^{-4\pi f_C\frac{T_s}{N}}}. \tag{10}$$

This equation is a complete form which yields exact results for all frequencies and under all component parameter values. As was seen in the main text, strong transmission occurs when the commutated transmission line media have significant capacitance. Furthermore, we are interested in the response in the vicinity of the commutation frequency as that is where our circulator operates. Therefore, to develop a better understanding of how our circuit works, (10) can be simplified under the assumptions of $f = f_s$ and $f_C/f_s = f_C T_s << 1$ or $C >> \frac{1}{2\pi f_s Z_0}$ as:

$$H_0^{V2,kernel}(f_s) \approx \frac{N(1-cos(\frac{2\pi}{N}))}{4\pi^2}e^{-j\pi/2}. \tag{11}$$

The overall transfer function $H_n^{V2}(f)$ from V_{in} to V_2 in Supplementary Figure 1 can be calculated by performing a summation across all paths [2] as follows:

$$\begin{aligned} H_n^{V2}(f) &= \sum_{l=0}^{N-1} H_n^{V2,kernel}(f)e^{-j2\pi nl/N} \\ &= NH_n^{V2,kernel}(f), n/N \in \mathbb{Z} \\ &= 0, n/N \notin \mathbb{Z}. \end{aligned} \tag{12}$$

This results in:

$$H_0^{V2}(f_s) = NH_0^{V2,kernel}(f_s) \approx \frac{N^2(1-cos(\frac{2\pi}{N}))}{4\pi^2}e^{-j\pi/2}. \quad (13)$$

Based on the definition of the scattering parameters, S_{21} can be computed as:

$$S_{21}(f_s) = 2H_0^{V2}(f_s) \approx \frac{N^2(1-cos(\frac{2\pi}{N}))}{2\pi^2}e^{-j\pi/2}. \quad (14)$$

A similar procedure may be followed to calculate S_{11}, requiring the computation of the transfer function from V_{in} to V_1 instead. In this case, $H_0^{V1,kernel} = H_{0,1}^{Vc,kernel}$, eventually resulting in

$$H_0^{V1}(f_s) \approx \frac{N^2(1-cos(\frac{2\pi}{N}))}{4\pi^2}. \quad (15)$$

Subsequently, S_{11} can be calculated as

$$S_{11}(f_s) = 2H_0^{V1}(f_s) - 1 \approx \frac{N^2(1-cos(\frac{2\pi}{N}))}{2\pi^2} - 1. \quad (16)$$

Similarly, by exciting the circuit with a voltage source at port 2 and performing a similar analysis, we can derive

$$S_{12}(f_s) \approx \frac{N^2(1 - cos(\frac{2\pi}{N}))}{2\pi^2} e^{j\pi/2}, \tag{17}$$

$$S_{22}(f_s) \approx \frac{N^2(1 - cos(\frac{2\pi}{N}))}{2\pi^2} - 1. \tag{18}$$

To summarize, the S-parameter matrix of the network shown in Supplementary Figure 1 at f_s under the assumption of $f_C/f_s << 1$ or $C >> \frac{1}{2\pi f_s Z_0}$ can be written as:

$$S(f_s) \approx \begin{bmatrix} \frac{N^2(1-cos(\frac{2\pi}{N}))}{2\pi^2} - 1 & \frac{N^2(1-cos(\frac{2\pi}{N}))}{2\pi^2} e^{j\pi/2} \\ \frac{N^2(1-cos(\frac{2\pi}{N}))}{2\pi^2} e^{-j\pi/2} & \frac{N^2(1-cos(\frac{2\pi}{N}))}{2\pi^2} - 1 \end{bmatrix}. \tag{19}$$

It can clearly be seen that S_{21} and S_{12} are reciprocal in magnitude response but are phase non-reciprocal. With -90° staggering, the phase of S_{21} is -90° while the phase of S_{12} is +90°. Furthermore, it can be easily shown that the magnitudes of S_{21} and S_{12} approach unity as $N \to \infty$ while the magnitudes of S_{11} and S_{22} approach 0. In other words, when $f_C/f_s << 1$ or $C >> \frac{1}{2\pi f_s Z_0}$, at f_s, strong signal transmission is seen across the staggered commutated network, particularly for large N, along with phase non-reciprocity. It should be mentioned that $N = 4$ and $N = 8$ are sufficient to ensure low loss transmission, as the magnitudes of S_{21} and S_{12} are -1.8dB and -0.45dB, respectively.

Supplementary References

1. Leung, B. *VLSI for Wireless Communication* (Springer, 2002).

2. Soer, M., Klumperink, E., de Boer, P.-T., van Vliet, F. & Nauta, B. Unified frequency-domain analysis of switched-series-RC passive mixers and samplers. *IEEE Transactions on Circuits and Systems I: Regular Papers* **57**, 2618–2631 (2010).
